# American Academy of Dental Science

**Published:** 1901-01

**Authors:** 

**Affiliations:** American Academy of Dental Science


### AMERICAN ACADEMY OF DENTAL SCIENCE.

THE thirty-third annual meeting and dinner of the American Academy of Dental Science was held at Young's Hotel, Boston, Mass., on Wednesday evening, November 14, at six o'clock, President V. C. Pond in the chair.

About thirty-five active Fellows were in attendance. The principal guests and speakers of the evening were Mr. George A. Littlefield, of Providence, R. I.; Rev. Charles J. Ketchum, assistant rector of St. Paul's Episcopal Church, Boston; and Mr. George Martin, supervisor of public schools in Boston.

Rarely has the Academy had the pleasure of listening to a more eloquent or interesting speaker at any of its annual gatherings than Mr. Littlefield proved himself to be. His address upon "American Citizenship" was a thoughtful and scholarly exposition of the subjective and collective characteristics of the ideal American of the present day, which, as he said, "it must be remembered, is a product of four hundred years of growth out of widely varied elements."

The speaker maintained that the first characteristic of American citizenship is based upon constitutional law, and that in the framing of our separate State constitutions, with such penalties as to compel their enforcement, and the interpretation and administration of these great charters, is to be found the chief historic differentiation of America from all other countries. Our forty-four State constitutions in writing all support and are each supported by the great key-stone of the written Constitution of the United States, each preserving perfect equipoise among the legislative and judicial functions; and the touchstone of all these great documents is the perfect equality of all men under them, without the slightest hereditary or aristocratic distinction.

The value of the religious motive and of education in promoting the growth of American character and manhood was forcibly demonstrated, as well as the lessons and experiences which have come to us from the Spanish-American War, and which have brought our national ensign into the boundless respect of all nations abroad.

Mr. George Martin was next introduced, and, following along the line of thought of the previous speaker, dwelt upon the relation of education in the public schools to the work of the various professions, maintaining that it is the aim of the public school to enable every child, whatever his inheritance may be,—material, intellectual, or social,—to get the most possible out of life and to put the most possible into life; in other words, that the strongest and best foundation for a professional life of any kind is necessarily laid in our public schools.

The Rev. Mr. Ketchum spoke entertainingly and in a general way upon the similitudes and the diversities of the clerical and the dental professions.

Previous to the banquet the following-named officers were chosen for the ensuing year: President, Dr. V. C. Pond; Vice-President, Dr. Frederick Bradley; Recording Secretary, Dr. Frank Perrin; Corresponding Secretary, Dr. George H. Payne; Treasurer, Dr. William Y. Allen; Librarian, Dr. Herman G. Hichborn; Editor, Dr. Charles H. Taft.

*Executive Committee.*—Drs. Forrest G. Eddy, Thomas Fillebrown, and Stephen G. Stevens.

CHARLES H. TAFT, D.M.D.,
*Editor American Academy of Dental Science.*

